# Supplementary material for: Electron aspirator using electron–electron scattering in nanoscale silicon
Source: Nat Commun. 2018 Dec 17;9:4813. doi: 10.1038/s41467-018-07278-8 (PMC6297221; doi:10.1038/s41467-018-07278-8)
Supplement: Supplementary file 1 — Supplementary Information [file 41467_2018_7278_MOESM1_ESM.pdf]

Supplementary Information

Electron Aspirator using Electron-electron Scattering in Nanoscale Silicon

Firdaus et al.

Supplementary Note 1. Basic characteristics of the device

Supplementary Figs. 1a and 1b show the collector- and emitter-gate characteristics, respectively, measured at 8 K with the substrate-gate voltage V_{SG} of – 15 V and the upper-gate voltage V_{UG} of 3.87 V. For the measurements of the emitter- (collector-) gate characteristics, the collector (emitter) gate was kept in OFF state.

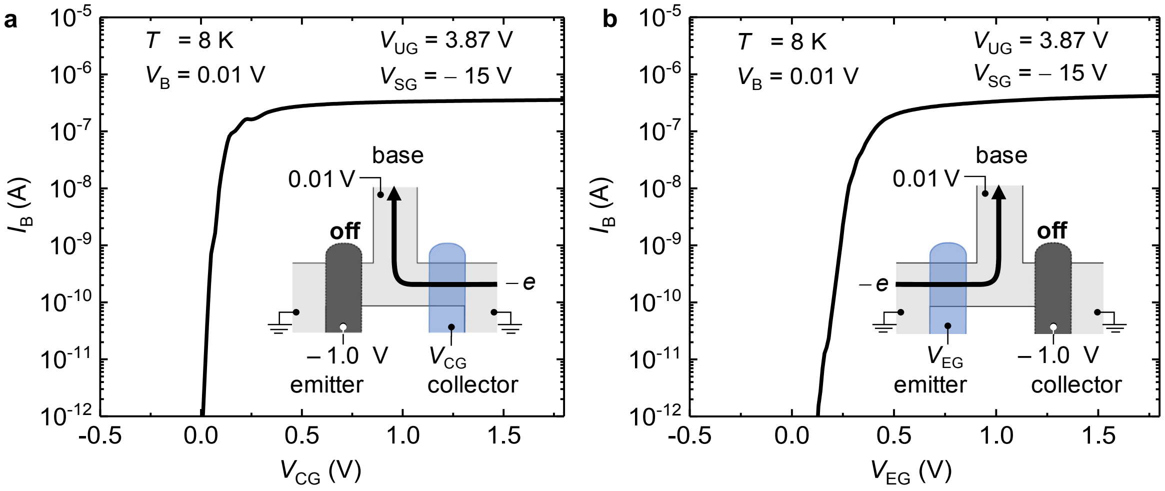

Supplementary Fig. 1. Basic characteristics of the device. a The collector-gate characteristics. **b** The emitter-gate characteristics. The measurement temperature is 8 K. The voltage setup is shown in each figure.

Supplementary Note 2. Expected potential profile of the device

In this note, we will show the expected potential profile in order to explain how electrons move in the device. We first remind the case where the electron transport is diffusive, and then we explain the case of the present device.

Assuming that the base and collector terminals have the same voltages V_0, and that the voltage at the intersection of the T-branch is V_{TB}, the voltage difference between the terminals and the intersection is $V_0 - V_{TB}$ for both terminals. In such a case, the currents are given by $I_B = R_B(V_0 - V_{TB})$ and $I_C = R_C(V_0 - V_{TB})$, where R_B and R_C are the resistance of the base-intersection and collector-intersection paths, respectively. In such a simple case, electrons move from the T-branch to each terminal if $V_0 - V_{TB} > 0$, or they move from each terminal to the T-branch if $V_0 - V_{TB} < 0$. Supplementary Fig. 2 shows these situations, where the profile of the electrostatic potential ϕ for electrons is drawn. Here, ϕ is defined as $\phi = (-e)V$, where V is the voltage and e (> 0) is the elementary charge. One can see that, in either case, the current polarity is the same, and the potential profile is symmetric with respect to the base and the collector terminals.

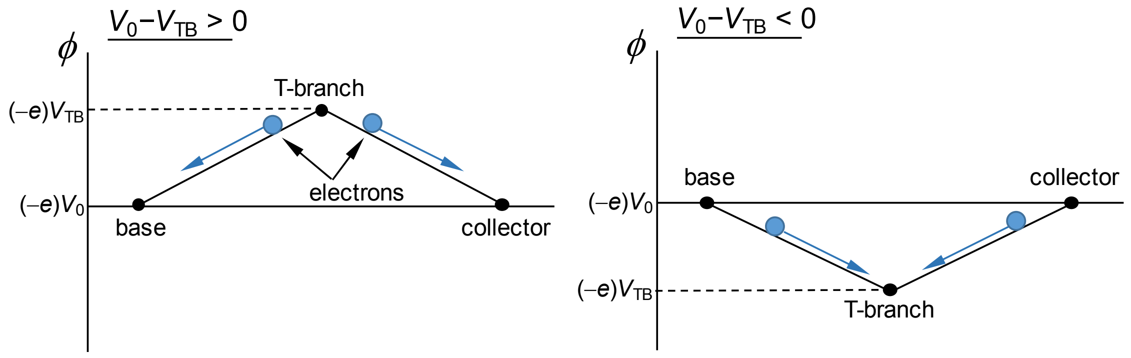

Supplementary Fig. 2. Profile of the potential ϕ and the electron flow for the case where the electron transport is diffusive. The voltages of the collector/base and the T-branch are denoted by V_0 and V_{TB}, respectively.

In the present device, on the other hand, the resistance of the collector channel R_C becomes negative in the aspirator operation. Supplementary Fig. 3 shows the expected profile of the electrostatic potential ϕ along the path between the base and the collector via the T-branch. Potential profile in the emitter channel is also shown by the dotted curve. (Note that the figures are not based on the calculation but merely conceptual.)

Supplementary Fig. 3a shows the case where the emitter voltage V_E is small. Nothing special happens and the current flows in a normal way like that shown in Supplementary Fig. 2 left. That is, electrons from the emitter are drained out either to the base or to the collector. The situation, however, drastically changes when V_E becomes negatively large. Supplementary Fig. 3b shows this situation.

Starting from the grounded base terminal, the potential ϕ first gradually decreases (due to a finite value of the base-channel resistance). At the intersection of the T-branch, we have a potential pocket, and then, the potential steeply increases underneath the collector gate, or at the exit of the T-branch to the collector. Finally, the potential again gradually decreases due to a finite value of the resistance of the collector channel, and turns back to zero.

As we have discussed in the main text, the potential pocket at the T-branch region is caused by the deficiency of electrons (or accumulation of positive charges) due to the *e*-*e* scattering. Electrons coming from the base gain the momentum and energy at the T-branch due to the *e*-*e* scattering, which allows these electrons to surmount the potential hump formed beneath the collector gate. As one can see in the figure, electrons are transferred from the T-branch region to the collector against the reversed bias (indicated by the red dashed line in Supplementary Fig. 3b), which in effect is the pumping operation. The energy for the pumping operation is supplied from the high-energy electrons injected from the emitter.

We here emphasize that the pumping effect at the T-branch region is equivalent to putting a battery, or a voltage source there (whose energy is supplied from the emitter electrons), and this is why we can obtain the electron flow between the two grounded terminals. Precisely speaking, we cannot define the

resistance in the T-branch-collector path, because I_C does not change linearly with $V_0 - V_{TB}$ there. However, we can still define the effective resistance R_C in the form of $I_C = R_C(V_0 - V_{TB})$, and based on this form, R_C becomes negative.

The origin of the asymmetric potential profile is the directional transfer of the emitter-electron momentum towards the collector. This is why the potential along the collector channel is always higher than that along the base channel.

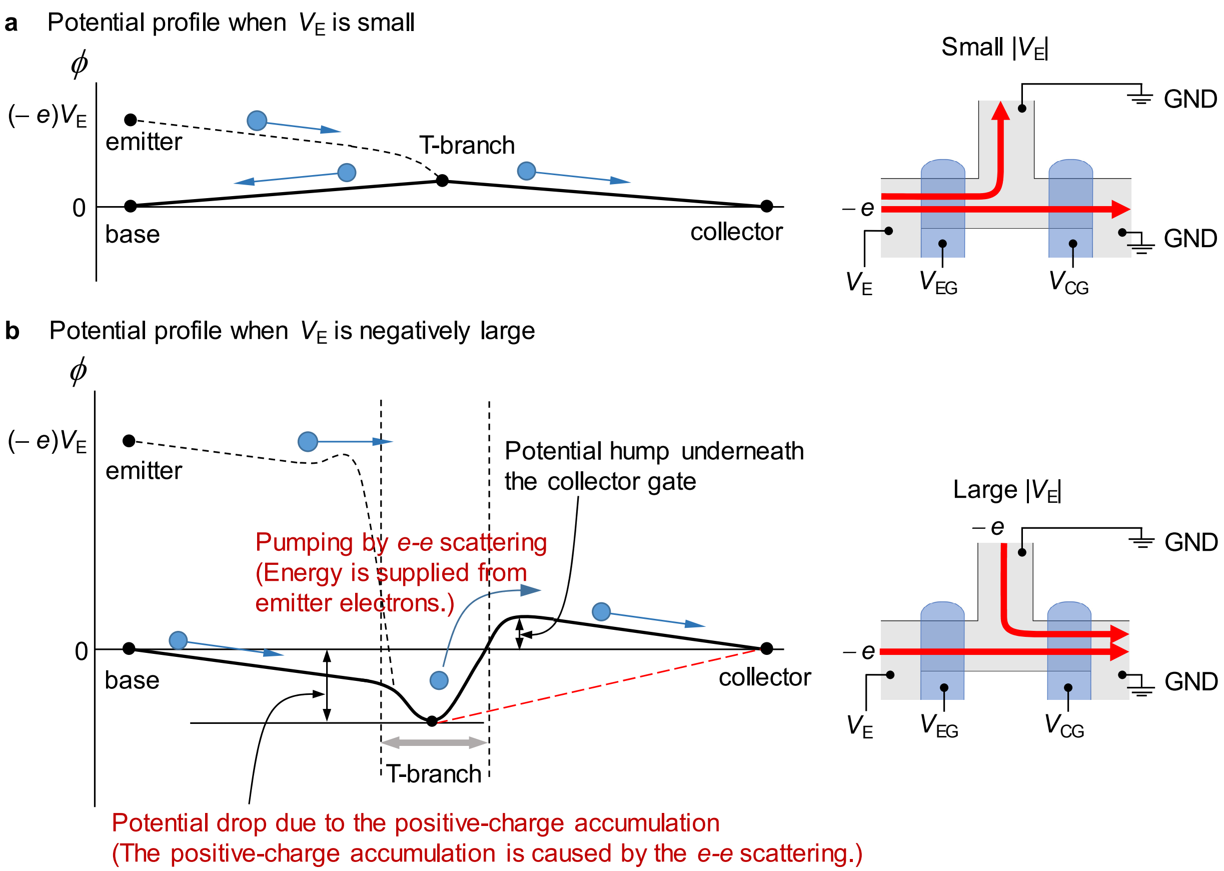

Supplementary Fig. 3. Potential profile and electron flow in the device. a For a small |V_E|. **b** For a large |V_E|.

Supplementary Note 3. Evaluation of E_F, τ_{ee} and l_{ee}

The Fermi energy E_F in the T-branch region was estimated from the electron density N and the density of states D. The value of N was estimated from $N = C_{OX} (V_{UG} - V_{UG\text{-}TH})$, where C_{OX} and $V_{UG\text{-}TH}$ are the gate oxide capacitance per unit area and the threshold voltage of the upper gate, respectively. The present front oxide is 20-nm thick. However, since the Si wire channel has the so-called gate-all-around structure, the concentration of the electric field makes the effective thickness $t_{OX\text{-}EFF}$ of the front oxide thinner than its physical value. We carefully considered this point and derived it from the $V_{UG\text{-}TH} - V_{SG}$ characteristics to be $t_{OX\text{-}EFF}$ = 12 nm. For $t_{OX\text{-}EFF}$ = 12 nm and $V_{UG\text{-}TH}$ = 0.7 V at V_{SG} = – 15 V, the N came to 5.7×10^{12} cm^{-2}. We kept this value for the measurements described in the main text.

The Si(100) two-dimensional electron channel is composed of the twofold and fourfold degenerate subbands with the twofold one having the lower energy (Supplementary Ref. 1). With $N = 5.7 \times 10^{12}$ cm^{-2}, it is a reasonable assumption that most of the cold electrons lie on the lowest (two-fold degenerate) subband when V_{SG} is negative (due to a deep triangular confinement potential) (Supplementary Ref. 2). In such a case, the Fermi energy E_F is simply given by N/D. The density of states D is given by $g_V m_D/\pi\hbar^2$, where g_V and m_D are the valley degeneracy and the density-of-state mass of the twofold degenerate subband, respectively, and $\hbar$ is the reduced Planck constant. From these values, E_F was estimated to be 36 meV.

In evaluating τ_{ee}, we employed the values for the lowest subband; $k_F = \sqrt{2m_D E_F}/\hbar$ and $k_{TF} = 2g_V m_D e^2/\kappa\hbar^2$, where κ is the averaged dielectric constant of Si and SiO_2 (Supplementary Ref. 1). In evaluating l_{ee}, we employed $l_{ee} = \tau_{ee} v_{IN}$, where v_{IN} is the forward velocity of the injected electrons. Since the injected electrons have a high energy of $|eV_E| + E_F$, they can be both in the twofold and fourfold degenerate subbands. Since the conductivity mass of the fourfold degenerate subband m_C' is larger than that of the twofold subband m_C, we took $v_{IN} = \sqrt{2(|eV_E| + E_F)/m_C'}$, giving shorter l_{ee}.

Supplementary Note 4. Comparison between the aspirator- and transistor-mode operations

In the upper panels of Supplementary Figs. 4a and 4b, we show, respectively, the transistor (gate-voltage) characteristics of the emitter, operating in the aspirator mode (the base is grounded) and in the transistor mode (the base is constant-current biased at I_B = 0 A). These figures correspond to Fig. 5c in the main text. The difference from Fig. 5c is that Supplementary Fig. 4 includes the emitter current I_E. In the lower panels, we show the sum of the three currents, $I_E + I_C + I_B$.

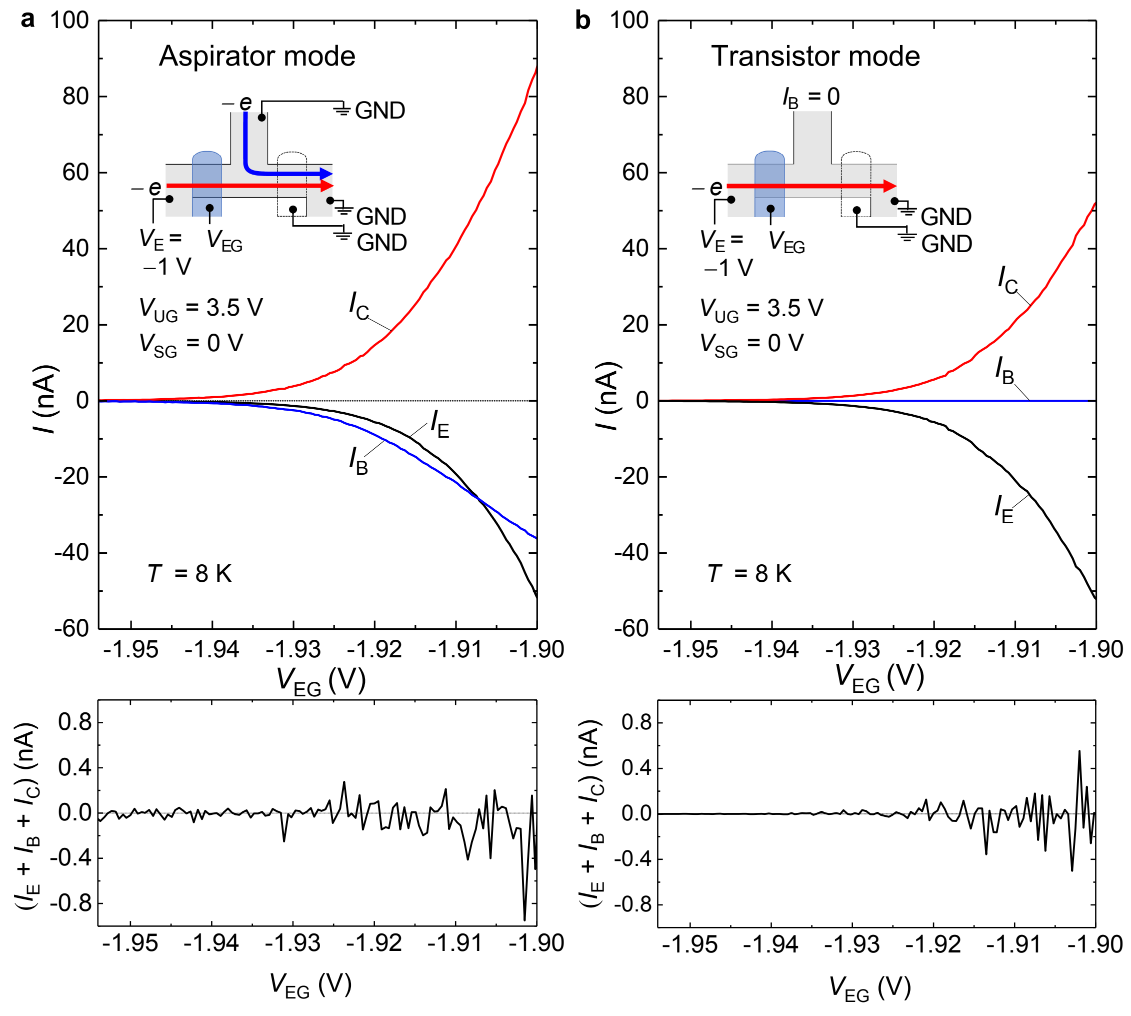

Supplementary Fig. 4. Comparison between the aspirator- and transistor-mode characteristics. **a** The aspirator-mode characteristics. **b** The transistor-mode characteristics. The lower panels show the sum of the three currents (emitter, collector, and base currents), $I_E + I_C + I_B$. The measurement conditions are the same as those shown in Fig. 5d of the main text.

Supplementary Note 5. Discussion towards the performance improvement

We here discuss the origin of the R_I reduction due to the increase in the $|I_E|$ (Fig. 5 of the main text) and due to the temperature (Fig. 1f of the main text). We will start with the analysis around Fig. 5a and 5c. As shown in these figures, the $R_{A/T}$ decreases when I_C exceeds about 2 – 3 nA. A simple explanation might be the insufficient electron density at the T-branch regions. This is because a low electron density results in a high resistance, which causes an unwanted potential drop at the T-branch region when the base electrons flow into there. This potential drop increases with $|I_E|$ and thus could reduce the number of electrons that can surmount the collector potential hump.

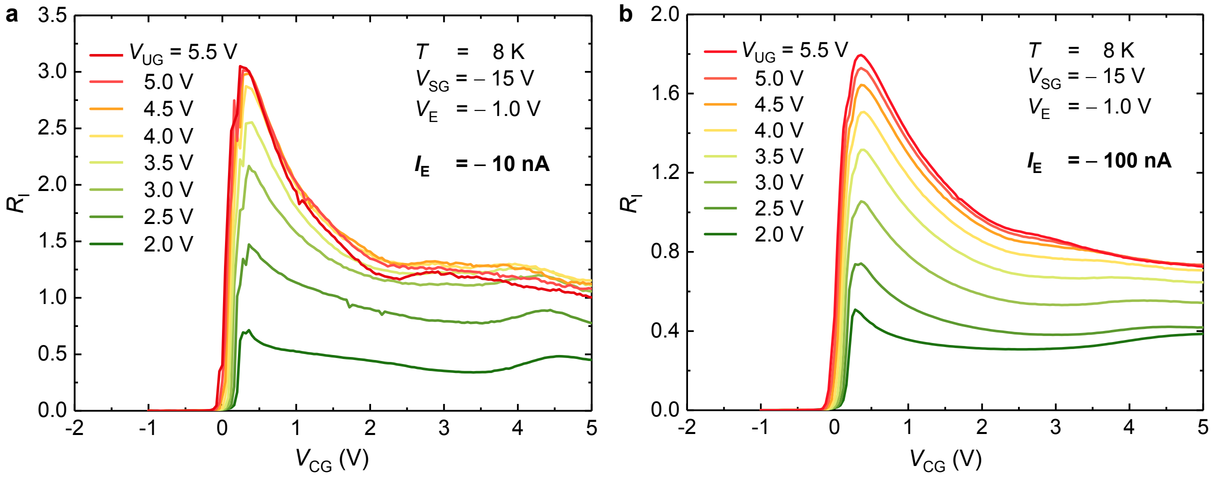

Supplementary Fig. 5. R_I as a function of V_{CG} using V_{UG} as a parameter. **a** The data for I_E = – 10 nA. **b** The data for I_E = – 100 nA. The measurement temperature is 8 K and the substrate-gate voltage V_{SG} and the emitter voltage V_E are, respectively, – 15 and – 1 V.

We will first show that the present results are unaccountable for by the above simple model. For this purpose, we investigated how the characteristics change when we vary the electron density at the T-branch region, for which we changed V_{UG} while keeping V_{SG} and V_E constant. Supplementary Fig. 5 shows the R_I as a function of V_{CG} using V_{UG} as a parameter for I_E = – 10 (a) and – 100 nA (b). The V_{SG} and V_E were fixed at – 15 and – 1 V, respectively. One can see that R_I increases with increasing V_{UG}, but it tends to saturate for both I_E = – 10 and – 100 nA. Supplementary Fig. 6 shows $R_{I\text{-}MAX}$ as a function of V_{UG} using I_E as a parameter. Each $R_{I\text{-}MAX}$ curve tends to saturate as V_{UG} increases. Noteworthy is that the saturation value is different for different values of $|I_E|$, decreasing with increasing $|I_E|$. This result excludes the

possibility of the insufficient electron-density model and indicates that the reduced $R_{I\text{-}MAX}$ is more fundamental and inherent for the present device.

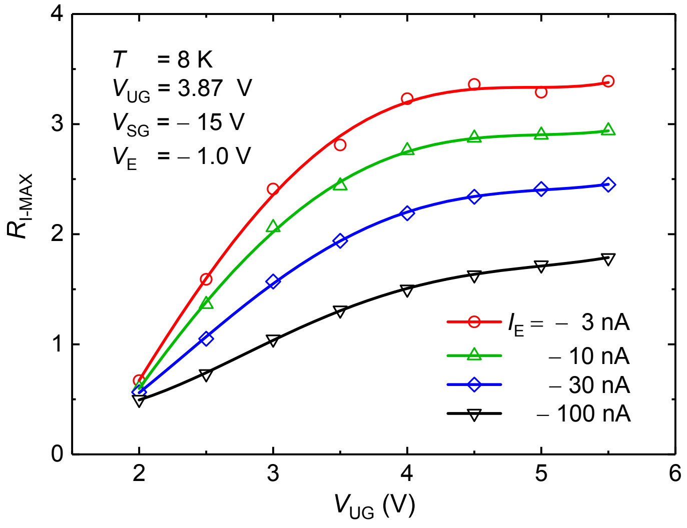

Supplementary Fig. 6. $R_{I\text{-}MAX}$ as a function of V_{UG} using I_E as a parameter. The curves are guides for the eyes.

A possible origin is the MN scattering. Suppose that one electron is injected into the T-branch region. As explained in the main text, the electron will encounter multiple *e-e* scatterings immediately after entering the T-branch region and transfer its momentum to other electrons. The secondary electrons will also transfer their momentum to other electrons, and such sequence continues until the electron system is thermalized with the phonon bath. The time scale of the relaxation with the lattice is on the order of 1 ps for Si (Supplementary Ref. 3). Therefore, if the injection current is, e.g., 1 nA (for which the averaged injection interval is on the order of 100 ps), the electron injection can be regarded as "one-by-one bombardment" to the Fermi sea. In such a case, each bombardment event is independent and the electron system reaches thermal equilibrium with the lattice (e.g., at 8 K) before the second electron is injected. Thus, R_I is independent of the injection current even in the presence of the MN scattering, which is what we observed for I_C less than about 2 – 3 nA in Fig. 5 of the main text.

When the injection current is increased, e.g., to 100 nA, the mean time-interval for the electron injection becomes comparable to the relaxation time, and the second electron is injected into the T-branch region before the electron system becomes thermalized with the lattice (at 8 K). In such a case, the electron

fluid becomes a stream, but we expect that this is the regime where the MN scattering severely degrades the current-enhancement capability. This is because the second and subsequently injected electrons would have more chances to collide with the back-scattered electrons, which decreases their forward momentum, degrading the current-enhancement capability. At the same time, the smaller mean time-interval for the electron injection as compared to the relaxation time results in the accumulation of energy, which could cause the increase in the local lattice temperature at the T-branch region. The increase in the lattice temperature then further degrades the current-enhancement capability due to the increase in the phonon scattering rate.

Based on the above consideration, we investigated the temperature dependence of the $R_{\text{I-MAX}}$ in order to judge which MN scattering process (interface roughness or phonon scattering) is dominantly responsible for the degradation observed in Fig. 5 of the main text. (As stated in the main text, the Coulomb scattering plays a minor role in the present device.) Supplementary Fig. 7 summarizes the results, where $R_{\text{I-MAX}}$ is plotted as a function of the substrate temperature T using I_E as a parameter. The voltage V_E was fixed at – 1.0 V.

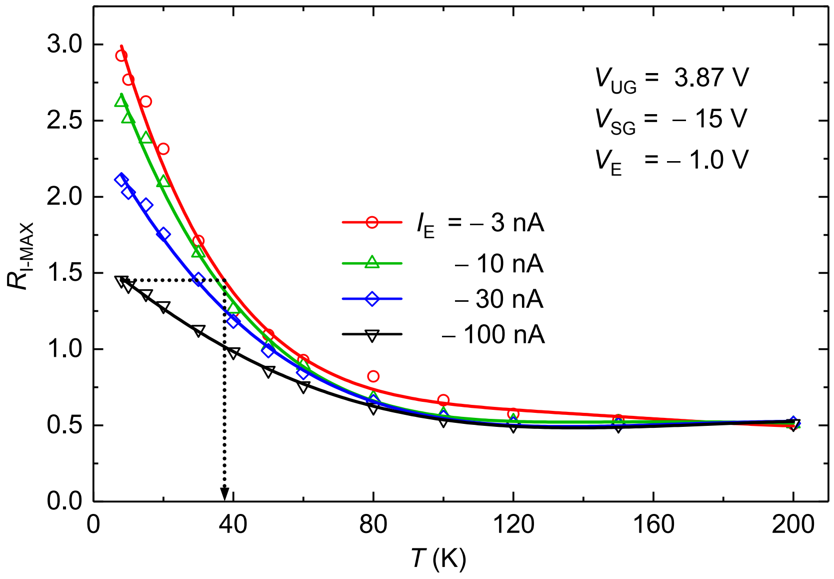

Supplementary Fig. 7. $R_{\text{I-MAX}}$ as a function of the substrate temperature T using I_E as a parameter. The curves are guides for the eyes.

One can see that the curves monotonically decrease even for I_E = – 100 nA. If the local lattice temperature increased due to the high power injection, the curve should be constant until the lattice temperature T reaches it. (For example, the curve should be constant for I_E = – 100 nA up to about 40 K, as indicated by the dotted line.) Therefore, the result indicates that the lattice temperature is not increased significantly even for I_E = – 100 nA, and the lower $R_{I\text{-}MAX}$ for higher $|I_E|$ at 8 K is not due to the increase in the phonon scattering rate.

In summary, the above analysis strongly suggests that the interface roughness scattering is dominantly responsible for the $R_{A/T}$ reduction with the increased I_C at 8 K and that the reduction of R_I at elevated temperatures is due to the phonon scattering.

As stated in the main text, the use of the SOI volume conduction and the atomically flat interfaces will be beneficial for relaxing the influence of the interface roughness. On the other hand, the phonon scattering is a more serious problem because it is expected to significantly deteriorate the current-enhancement capability due to its isotropic nature. (The phonon scattering angle is random both for high-energy inter-valley and low-energy acoustic phonons (Supplementary Ref. 4).) A most direct way to avoid this will be to make the device smaller than the phonon scattering length L_{PH} ($L < L_{PH}$). This will also be beneficial for the R_I enhancement because more electrons can pass the collector gate due to less amount of energy loss.

Supplementary Note 6. Accuracy of the input voltages

Supplementary Fig. 8a shows the voltage setup for the measurement of the current flowing between the base and the collector when both terminals are grounded. The emitter gate was set in OFF state by applying a negative voltage (V_{EG} = – 1.5 V). Then, in order to avoid any leakage current from the emitter terminal, it was constant-current biased at I_E = 0 A. On the other hand, the collector gate was set in ON state by applying a positive voltage (V_{CG} = 1.5 V). This voltage is large enough to make the channel beneath the collector gate being in strong inversion. The upper gate voltage was set at V_{UG} = 3.5 V, as in the case of the aspirator experiments shown in Figs. 5c and 5d of the main text. The above voltage setup enables us to measure the current flowing between the base and collector without disturbance by the emitter current.

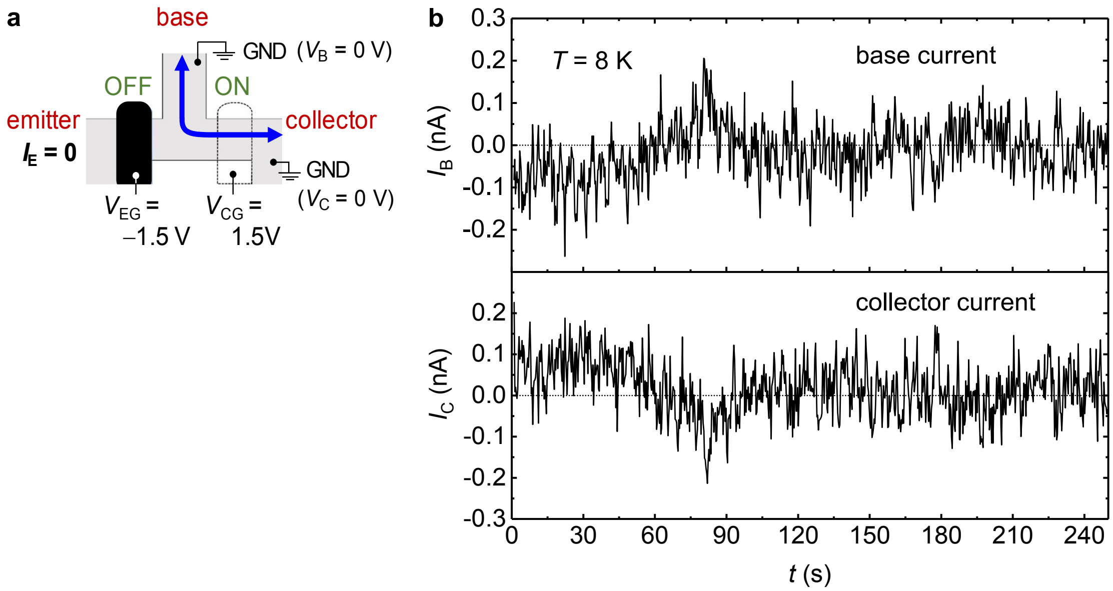

Supplementary Fig. 8. Offset and fluctuation of the base and collector currents, I_B and I_C, as a function of time. a Voltage setup. **b** Experimental results. Both base and collector terminals are grounded. Measurement temperature is 8 K.

Supplementary Fig. 8b shows the base and collector currents, I_B and I_C, measured after the calibration of the SMUs used, as a function of time with the base and collector voltages V_B and V_C both set at 0 V. The measurement temperature is 8 K. One can see that both I_B and I_C have no offset and the fluctuation is about ± 200 pA. This current fluctuation is much smaller than the typical value of the current used for the aspirator experiments, which is on the order of 10 nA.

Supplementary Fig. 9a shows the voltage setup for the second type of measurements. In these measurements, we swept the base voltage V_B keeping the collector grounded (V_C = 0 V), and measured the I_B and I_C characteristics. We performed 60 scans in order to check the fluctuation, and the results are shown in Supplementary Fig. 9b. One can see that the conduction is ohmic in the measured voltage range, and the resistance was estimated to be about 3×10^4 Ω. Supplementary Fig. 9c shows the magnified view of Supplementary Fig. 9b around V_B = 0 V. One can see in the upper panel that the current fluctuation of ± 250 pA corresponds to the voltage fluctuation of about ± 8 μV. In other words, the voltage difference between the base and collector is zero with an uncertainty only of the order of 10^{-5} V.

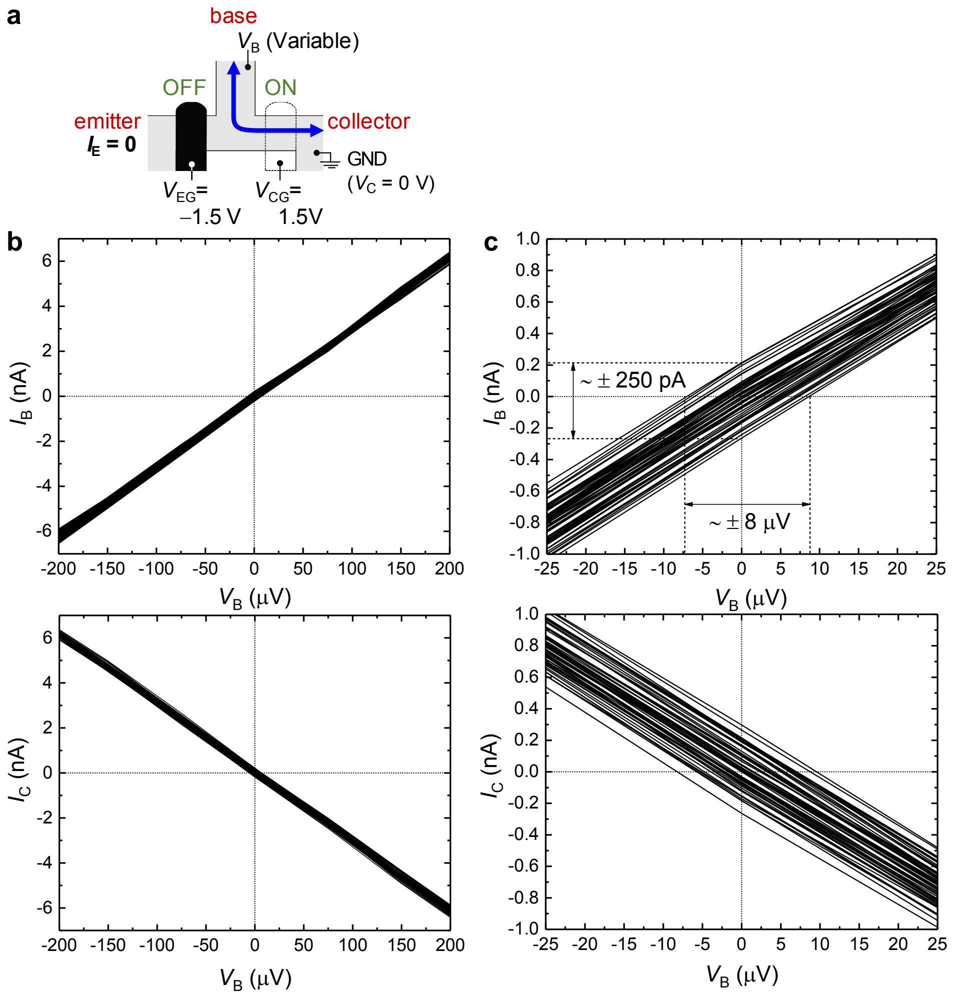

Supplementary Fig. 9. Base and collector currents, I_B and I_C, as a function of base voltage V_B. a Voltage setup. **b-c** Experimental results. Collector terminal is grounded. Measurement temperature is 8 K.

From the above results, we conclude that, with both the base and the collector terminals grounded, the voltage difference between these terminals is negligibly small, and no unintentional voltage is generated between the base and the collector.

Supplementary References

[1] Ando, T., Fowler, A. B., Stern, F. Electronic properties of two-dimensional systems. *Rev. Mod. Phys*. **54**, 437-672 (1982).

[2] Takashina, K. *et al*. Intersubband scattering in double-gate MOSFETs. *IEEE Trans. Nanotechnol*. **5**, 430-435 (2006).

[3] Goldman. J. R. & Prybyla, J. A. Ultrafast dynamics of laser-excited electron distributions in silicon. *Phys. Rev. Lett.* **72**, 1364-1367 (1994).

[4] Herring, C. Transport properties of a many-valley semiconductor. *Bell System Tech. J.* **34**, 237-290 (1955).
